# Editorial: Membrane Lipids in T Cell Functions

**DOI:** 10.3389/fimmu.2018.01608

**Published:** 2018-07-09

**Authors:** Loretta Tuosto, Chenqi Xu

**Affiliations:** ^1^Department of Biology and Biotechnology Charles Darwin, Sapienza University, Rome, Italy; ^2^State Key Laboratory of Molecular Biology, Chinese Academy Center for Excellence in Molecular Cell Science, Shanghai Institute of Biochemistry and Cell Biology, Chinese Academy of Sciences, University of Chinese Academy of Sciences, Shanghai, China; ^3^School of Life Science and Technology, ShanghaiTech University, Shanghai, China

**Keywords:** lipid rafts, phosphatidylinositol 4-phosphate 5-kinases, inositol (1,3,4,5) tetrakisphosphate, cholesterol metabolism, glycosphingolipid metabolism, neutral sphingomyelinase, membrane charges

**Editorial on the Research Topic**

**Membrane Lipids in T Cell Functions**

Plasma membrane lipids play essential roles in regulating T cell signaling, differentiation, and effector functions. The major lipid species in the plasma membrane are glycerophospholipids, sphingolipids, and sterol lipids. TCR and costimulatory molecules lead to profound changes in the composition, distribution, and dynamic of plasma membrane lipids. For instance, cholesterol, sphingomyelin, and saturated phosphocholine are enriched at the contact zone between T cells and antigen-presenting cells during peptide/MHC complexes recognition, where they constitute a platform of lipid domains essential for optimal T cell signaling. Glycerophospholipid provide docking sites for binding pivotal signaling proteins as well as for their conformation, portioning, and mobility. Finally, plasma membrane lipids also act as second messengers with important immune-regulatory functions.

This Research Topic contains seven articles that review the current understanding of the mechanisms and molecules involved in the metabolism and function of membrane lipids and how differences in their content may affect T cell functional properties.

One of the main relevant discoveries in T cell biology has been the identification of specific signaling platforms enriched of cholesterol and glycosphingolipids, named lipid rafts, where critical enzymes, adaptors, and scaffold proteins are accumulated and trigger pivotal signaling pathways (1, 2). After a nice historical revision of the biochemical, biophysical, and imaging approaches that have been exploited during the past 20 years for identifying the composition, dynamic, and functions of lipid rafts, Zumerle et al. discuss the relevance of membrane rafts in T cell signaling functions and the pivotal role of CD28 costimulatory molecule in clustering membrane rafts at the immunological synapse (IS) through massive actin-reorganization events. Interestingly, cytoskeleton reorganization and actin polymerization are also regulated by phosphatidylinositol 4,5-biphosphate (PIP2), a membrane phospholipid that controls the activity of several proteins important for T cell functions (Porciello et al.). Phosphatidylinositol 4-phosphate 5-kinases (PIP5K) are mainly involved in PIP2 synthesis and, in human, three isoforms (α, β, and γ) and further splice variants have been identified. PIP5Kα and β have been recently identified as the main regulators of CD28 inflammatory and costimulatory functions (3–5), thus representing useful targets for inflammatory and autoimmune-based diseases. Using a recently discovered inhibitor of PIP5Kα, ISA-2011B (6, 7), Kunkl et al. evidenced a critical role of PIP5Kα in regulating CD28-mediated upregulation of inflammatory cytokines in type 1 diabetes patients.

Another important function of PIP2 is to serve as a substrate of class 1 phosphoinositide 3-kinases (PI3K) that, by phosphorylating PIP2 on the D3 position of the inositol ring, generates phosphatidylinositol 3,4,5-triphosphate (PIP3) lipids. The PI3K signaling pathway is crucial for the development, maturation, and function of immune cells and, in their review, Elich and Sauer nicely discuss the role of PIP3 analogs, IP4 and IP7, and the enzymes responsible for their synthesis and turnover on stem cell homeostasis, neutrophil, and NK cell function as well as development and function of B cells and T cells. The pathological role of deregulated IP4 activity in lymphocytes is also discussed as well as its association with some B cell malignancies such as Kawasaki disease or severe combined immunodeficiency.

The maintenance of lipid raft structure and stability in T cells is also ensured by the amount of cholesterol (8), since changes in cholesterol content may affect the biophysical properties of lipid rafts (9). The influence of cholesterol transcriptional regulators, in particular liver X receptors (LXRs), estrogen receptors (ERs), and peroxisome proliferator-activated receptors (PPARs), on membrane lipid rafts and T cell functions is discussed in the review by Robinson et al. who describe the role of these nuclear receptor transcription factors in manipulating lipid metabolism. In particular, the authors highlight the implication of deregulated LXR, ER, and PPAR functioning in several immune-based diseases and the potential benefit of targeting these receptors as a therapeutic approach for autoimmunity and inflammatory diseases.

Given the critical structural and functional roles of cholesterol, cholesterol metabolism of T cells is of high interest for both basic and translation researches. In the review by Bietz et al. the authors discuss recent seminal works showing that cholesterol metabolism is crucial not only for making lipid building blocks in proliferating T cells but also for the acquisition of T-cell effector function (10–12). Modulating cholesterol metabolism can thus be used to harness T-cell activity in different disease contexts such as cancer and autoimmunity. Taking the advantage of drugs previously developed for metabolic diseases, researchers can repurpose them for immunotherapies, which opens a new venue for potential clinical applications. The Yin-and-yang concept can be well reflected in the therapeutic designs.

The biophysical properties of cellular membrane are also regulated by sphingomyelinases that, by catalyzing the breakdown of sphingomyelin into ceramide, play critical roles in actin cytoskeleton reorganization and polarization of T lymphocytes (13–15). Using both inhibitory drugs (ES048) and specific siRNA, Collenburg et al. evidence that neutral sphingomyelinase activity is required for CXCR4-induced cytoskeleton reorganization, the polarization and directional migration of CD4^+^ T lymphocytes as well as for LFA-1 affinity maturation and adhesion during trans-endothelial migration.

One novel aspect discussed by Ma et al. is the impact of membrane charges on TCR signaling. For instance, the plasma membrane has an asymmetrical distribution of phospholipids with neutral phospholipids such as sphingomyelin located primarily in the outer leaflet and anionic phospholipids at the inner leaflet (16). The authors focus on the role of electrostatic potential of the inner leaflet in regulating the early signaling events triggered by TCR and its association with the cytoplasmic tails of CD3. They also discuss the influence of charged lipids within the IS in creating an electrostatic potential that is differentially regulated from the rest of the plasma membrane and that is essential for efficient TCR signaling.

In summary, we are still at the early stage of understanding the sophisticated roles of membrane lipids in T cell biology, but the current progresses highlight the bright future of this field. Understanding the structural and functional roles of diverse lipid molecules in T cells will lead to better understanding of T cell biology and also enable developments of novel immunotherapies against different diseases.

## Author Contributions

LT wrote the first draft of the manuscript and updated the last version. CX completed and corrected the draft.

## Conflict of Interest Statement

The authors declare that the research was conducted in the absence of any commercial or financial relationships that could be construed as a potential conflict of interest.
